# Educational needs assessment among 10–14-year-old girls about puberty adolescent health of Ardebil

**DOI:** 10.1186/s13690-019-0388-3

**Published:** 2020-02-03

**Authors:** Arash Ziapour, Manoj Sharma, Nazila NeJhaddadgar, Afrouz Mardi, Sedigheh Sadat Tavafian

**Affiliations:** 1 Health Education and Health Promotion, Health Institute, Kermanshah University of Medical Sciences, Kermanshah, Iran; 2 Behavioral & Environmental Health, School of Public Health, Jackson State University, Jackson, MS USA; 3 Health Promotion and Education, Department of Health Promotion and Education, Ardabil University of Medical Sciences, Ardabil, Iran; 4 Reproductive Health, School of Health, Ardabil University of Medical Science, Ardabil, Iran; 5 Health Education and Health Promotion, Tarbiat Modares University, Tehran, Iran

**Keywords:** Adolescent health, Adolescents, Educational needs, Girls

## Abstract

**Background:**

This study aimed to investigate the educational needs of 10–14-year-old girls about adolescent puberty health in Ardabil City in Iran, the matter of adolescent puberty health is one of the most important health priorities, especially for girls.

**Methods:**

This cross-sectional descriptive-correlational study was performed on girl students through stratified-cluster sampling in Ardabil city. The data gathering tool was a self-reported researcher-designed questionnaire consisting of 10 demographic questions and 35 self-care questions in 5 domains of self-care awareness, self-efficacy, enablers, enhancers and behaviors, based on the reviewed articles and resources. Descriptive statistics and chi-square test, correlation coefficient and regression were used to analyze the quantitative data.

**Results:**

The mean age of the girls was 12.98 ± 4.67. There was a significant relationship between the mother’s level of education (*p* < 0.001) and students ‘knowledge about puberty and between mother’s age (*p* < 0.002) and students’ awareness of self-care behaviors during adolescence. The results of this study showed that 73.8% of girls were aware of puberty and menstruation problems. About 74.3% of girls had poor self-efficacy. About 77% of female students in the study did not have access to the required educational resources and classes that are one of the most important enablers of behavior formation. Eighty-eight percent of the families did not talk about the hygiene practices related to their children. The most common source of information about maternal health related practices was from mothers (64.8%). Correlation test between adolescents’ health behavior and awareness (r = 0.12 *p* < 0.007) and between self-efficacy and health behaviors (r = 0.14, *p* < 0.001) revealed significant and positive relationships.

**Conclusion:**

There is a need among adolescent girls for information about adolescent health and related health behaviors. Currently there is lack of adequate and accurate information. The role of mothers is the most important source of information for adolescent girls and educational approaches for mothers and adolescents should be designed.

## Background

Adolescence is defined as the age group of 10–19 years according to the World Health Organization [1]. Adolescence is one of the best and most valuable years of life, because it is the beginning of physical, psychological and social change and is a critical period in which puberty occurs [2]. Today, More than 50% of the world’s population is under the age of 25, with one fifth (half a billion) of them being teenagers [1, 3]. Over 20% of the population in Iran is adolescents aged 10 to 19 years and over half of the population is under 25 years of age [4]. The first symptom of adolescence is sexual puberty [5].

Puberty is the most important turning point in adolescence, which is the transition period from childhood to adulthood and the time to gain fertility. This period is caused by the activation of the sexual glands and is accompanied by a series of symptoms and physical and mental changes [3, 6]. How to deal with puberty and the puberty process are important issues that need to be addressed by the authorities and parents. When children become adolescents, they confused with their physical changes and in most cases families become helpless due to their disability to train for puberty [7]. Studies have shown that adolescents, families, and most trainers do not have enough information about the normal process of puberty and the characteristics of adolescence [8]. So many physical, mental and emotional problems may be rooted in the health of this period. Adolescence forms the different period of one’s life [9].

Today, attention to adolescent health is an important issue The World Health Organization has also identified adolescent girls’ educational needs as a priority [10]. The International Population Conference has also called on the committed nations in a declaration to identify and address these needs [11]. The adolescent may not be trained as an inefficient force due to the lack of attention to the physical and psychological educational needs of the period [12]. Also, the ambiguity, attention, and anxiety of adolescents about the process of puberty influence their perception of their values [13, 14]. Studies show that 47.9% of adolescents lacked education and information about puberty and 39.9% of them had confusion and disgust at the early stages of puberty [15]. Another study conducted by the Iranian Ministry of Health and Medical Education in Tehran showed that more than half of 10–14-year-olds do not know about the symptoms of puberty or information is incomplete about symptom of puberty [16, 17]. Also based on Olfatis study; girl students’ knowledge of puberty healthy behaviors was very low. This is due to the lack of proper transmission of information from parents to their daughters, while addressing to common problems of puberty ensures the generation health [9, 18]. Families and school educators frequently do not able to respond adolescent’s issues and problems during puberty. And their information is not adequate about the common problems of girls during puberty, sometimes families do not provide a definition of the puberty, adolescence and health of this period to their children, while adolescent girls’ health issues are not only relevant to themselves but also because they are mothers of tomorrow, it is also beneficial to the family, community and future [19].

According to the cited issues and the importance of this period in the life of the future and the beginning of physical, mental and sexual changes we intended to carry out a research aimed at determining the educational needs of adolescent girls in Ardebil, to make a step towards raising the level of consciousness and culture of mothers of future of our country and helps to health authorities for develop educational programs to raise girls’ awareness of issues related to puberty and health behaviors.

## Methods

### Study design

This correlation- Cross-sectional descriptive study was conducted on 452 girls student (10–14 years old) in Ardabil (Ardabil is an ancient city in northwestern Iran, and the capital of Ardabil Province). The city was first divided into five regions: north, south, east, west and central, and then randomly selected a number of clusters (schools) proportional to the population of each region. The sample size was determined based on inclusion criteria and 95% confidence level and 80% test power. Inclusion criteria include: 10–14 years old, menarche experience, residence in Ardebil city, would like to participate in the study. And exclusion criteria, including those under 10 and over 14 years old. Inexperienced Menarche, not wanting to take part in the study.

For this study, a self-care puberty researcher-made questionnaire was used. The validity of questionnaire was determined by two methods of face validity and content validity, which confirmed by experts of health education, psychology, reproductive health. The total content validity index (CVI) in the “relevancy”, “simplicity”, and “clarity” respectively equals 82.6, 92.9, and 90.7. The reliability of the questionnaire further evaluated through internal consistency (α = 0. 83) and test-retest (r = 0.82).

This questionnaire consisted of two parts. The first part consisted of 10 questions on demographic and family information and the second part consisted of 35 questions in 5 domains of awareness, self-efficacy, enablers, enhancers, and self-care behaviors.

The questions were divided into 5 sections: Questions 1–14 were related to students’ awareness of the puberty of their self-care behaviors. In this section, each correct answer was scored with 1 and the score of the wrong answer with zero points. Maximum awareness score was set at 14 and the minimum score was zero.

Questions 15–17 assessed adolescents’ self-efficacy (judging and believing in their ability to perform tasks and activities) related on their ability to perform self-care behaviors. The questions were rated on a 4-point Likert scale of 4 I’m quite sure. From adolescents were asked to rate their confidence by choosing one of the options for adherence of adolescent self-care behaviors. Each correct answer had 4 points, with a maximum score of 12 and a minimum score of 4.

Behavioral enablers included questions from 18 to 19 on teaching materials, training courses and counseling were available with the answer yes and no. And it was scored zero and one. The maximum score in this section was set at 2 and at least zero.

Questions 20–23 also examined behavioral enhancers (supporters and self-care behavior) with two-choice questions with yes-and-no answer. In this section, the score zero and one. The maximum score in this section was 4 and at least zero.

The plan was first approved by the university’s research vice-president and received Code of Ethics 1397.064. And Implementation permits were obtained from the provincial education authorities. Then data were collected after obtaining parent consent and assurance that the researcher’s confidential information was not disclosed within 2 months. The data were analyzed by SPSS software version 19 and analyzed using chi-square, correlation coefficient and regression tests.

### Ethical consideration

The plan was first approved by the university’s research vice-president and received Code of Ethics 1397.064. And Implementation permits were obtained from the provincial education authorities. Then data were collected after obtaining parent consent and assurance that the researcher’s confidential information was not disclosed within 2 months. The data were analyzed by SPSS software version 23 and analyzed using chi-square, correlation coefficient and regression tests.

## Results

In this study, 452 first-year high school girls were studied with a mean age of 12.98 67 4.67 years, 61.1% of participants were first and second children, and their mean menstrual age was 11.21 ± 1.68. 39.1% of girls were obese or overweight. Other demographic and social characteristics of the samples under study are shown in Table 1.

**Table 1:** Demographic characteristics

Variable	Frequency (Percent) Number
Father’s age (mean and standard deviation)	49.68 ± 7.77
Father’s education	120 (25.3) Under the diploma
	354 (74.7) Academic
Mother’s age (mean and standard deviation)	46/46 ± 7.35
Mother’s education	21 (4.4)illiterate
	130 (27/4) Under the diploma
	323 (68.1) Academic
Birth Rank	323 (68.1) 2–1
	134 (28.3) 5–3
	17 (10.6) 6<
Household income	225 (47.5) Millions 2<
	190 (40.1) Millions 5–2
	59 (12.4)5<

Chi-square test showed a statistically significant relationship between maternal education level (*p* = 0.001) and students ‘awareness of puberty and between maternal age (*p* = 0.002) and students’ awareness of adolescent self-care behaviors. 78% of students and 87.65% of parents were interested in attending adolescents’ special education classes.

The results of this study showed that 73.8% of girls had poor knowledge about puberty and menstruation. According to prioritizing questions 5,8,7,11 and 6 about puberty and its symptoms, food pyramid and its effect on health, premenstrual syndrome the adolescent girls’ calorie requirement during puberty, menstrual age, respectively, had the lowest score and were ranked the top 5 educational priorities in the awareness domain (Table 2).

**Table 2:** Adolescent girls’ awareness score status

Row	Awareness questions	SD ± Average
1	What are the stages of girls’ puberty, respectively?	1.4 ± 0.86
2	Importance of adolescent girls’ familiarity with the food pyramid Which of the following is true?	1.91 ± 0.97
3	What is Prenatal Month Syndrome and What are the Symptoms?	1.99 ± 0.90
4	Adolescent girls need a few kilograms of energy during adolescence?	2 ± 1.03
5	At what age do girls’ first monthly habits appear?	2.05 ± 0.80

*SD* standard deviation

Results showed that 74.3% of the girls had poor self-efficacy and based on 30, 29, 28 prioritization, three priorities of educational intervention were self-efficacy. In regular daily exercise, a balanced diet and self-care behaviors during puberty are not trusted (Table 3).

**Table 3:** Adolescent girls self-efficacy score status

Row	Behavioral questions	SD ± Average
1	To my ability to exercise regularly for 30 min a day…	1.77 ± 0.42
2	To my ability to maintain a healthy diet…	0.85 ± 0.34
3	To my ability to observe adolescent self-care behaviors…	2.92 ± 1.28

*SD* standard deviation

Findings showed that 77% of female students did not have access to the educational resources and training needed were one of the most important enablers of behavior formation. 89% did not have adequate exercise space for physical activity.

And 78.3% of families did not talk to their children about puberty. However, 64.8% of students tended to talk to their family members, especially mothers, and secondarily to friends and peers about the puberty of health-related practices. Female students who participated in the study of healthy eating patterns also found 51.1% of family members and 35.4% of friends and peers to be the most important in their food choices. In this study, 50.4% of the students had more sandwich knowledge than their other fast food priority and had a weekly meal with their friends or family members at least once a week.

In this study, self-care behavior was 80.4% of adolescents in the medium study and based on 19, 12, 22, 20 and 15 5 priorities of intervention., How to use the bar for changing the health bar, breakfast meal choices and menstrual hygiene. Also, most of the adolescents who participated in the study of the most important adolescents’ moods and morals considered the sense of independence and inclination to the opposite sex (Table 4).

**Table 4:** Adolescent girls self-care behavior score status

Row	Awareness questions	SD ± Average
1	Do you bathe during your monthly period?	1.76 ± 0.42
2	How many shares of dairy do you consume daily?	2.22 ± 0.63
3	How often do you change your health bar for use during your monthly routine?	2.54 ± 0.97
4	Which of the following foods do you typically use in your breakfast program?	2.60 ± 0.64
5	How many times a day do you change your underwear during your menstrual period?	2.71 ± 0.82

*SD* standard deviation

According to the findings, promoting self-efficacy was the most important educational priority in the formation of healthy eating behavior. According to the correlation test between the health behavior of adolescence and awareness (r = 0.12, P < 0.007 and between health behavior and self-efficacy) (r = 0.14, P < 0.001 among adolescent girls was significant and positive, and based on linear regression by backward elimination among the variables with positive correlation with self-efficacy behavior was the most important predictor of adolescent behavior in this study. (Table 5).

**Table 5:** Regression test results

Variable	Non-standard coefficients		Standard coefficient	t	*P*-value
	B	The standard error	Beta		
Efficacy	0.14	0.48	0.13	2.91	0.004

## Discussion

The results showed that adolescent girls should have educational needs and information about puberty and its symptoms, food pyramid and its effect on health, calorie requirements of adolescent girls, premenstrual syndrome, and menstrual age while they do not have enough information about puberty. Despite the tendency of girls to become aware of puberty, the majority of girls still did not know the full meaning of puberty and its changes. The girls also had a false and untrue scientific and physiological awareness of the phenomenon of puberty, which is due to incomplete information or negative beliefs about puberty, and consistent with the results of the Dabiri and Darabi studies [8, 20]. And because of the unwillingness of parents, teachers, and school health educators to inform teens about information about puberty. Mothers are more likely to want their daughters unaware of reproductive issues. And sometimes they find these things unpleasant and embarrassing [21, 22]. The results of this study are in accordance with the findings of Simber et al. In Tehran, which showed poor health awareness during adolescence [23]. Graaf’s results in India also show that girls ‘awareness of puberty is dispersed and heterogeneous and plays a role in controlling attitudes in girls’ lack of information [23], which has been effective in girls’ lack of information [24]. Therefore, puberty health education should start from the lower age groups. Unfortunately, being unaware of changes in adolescence, reproductive health and marriage usually lead to unwanted pregnancies, STDs and the bad consequences of failing to comply with health tips [8]. obscure for adolescents, and if they hear ambiguous information not only disturbed, but often frightened. And if they unable to answer for questions and needs, they refer to peers and incomplete sources for information [8]. It is therefore important to be aware of puberty questions and educational needs. This awareness may be presented effectively in educational materials. Providing educational materials will be a good solution for families and adolescents. In addition, there was a significant relationship between maternal education and puberty awareness. The results of this study showed that with increasing parental literacy, adolescent girls’ awareness of puberty and related health behaviors increases, which is in line with Tazeen et al. [25]. Of course, high parental awareness and information affect children’s thinking, perception, and attitude. The adolescent perspective is promoted with a positive and realistic attitude to the issues of puberty. Educated parents may have different attitudes toward their children than those who do not have enough education, On the other hand, the illiterate are likely to pay less attention to their own health and that of their children and cause premature marriage and pregnancy complications during puberty. In this study, as mothers aging, girls’ awareness of puberty issues increases, perhaps due to the increasing age of their parents, their child-related information and experience and subsequent age-related education. In this study, mothers were the most important source of information on puberty health, thus reinforcing the appropriate relationship between mothers and their adolescent daughters and removing communication barriers such as failure to express maturity information or even mothers’ indifference to its health consequences and consequences. It should be included in the mother’s education program. To this end, they need to familiarize them with the physical, psychological, mood and behavioral changes of puberty and prevent the negative consequences of this period [8].. In this study, peers and friends were other sources of information that could have dangerous consequences. Many families believe that discussing puberty and sexuality is a form of rubbish. Adolescents are sensitive to such issues and are looking for information about them. And choose the easiest ways (including friends, peers, etc.) that sometimes misleading information from friends and other sources can cause sexual deviations and problems for the individual and society, but the role of professionals, Schools, educators and institutions should not be neglected in educational planning [26]. Self-efficacy in adolescent self-care behaviors, girls’ continued regular daily exercise, balanced diet, and self-care behaviors during puberty were poor in this study.

People with higher self-efficacy are more likely to find themselves more motivated to continue to behave in the face of obstacles [27]. On the other hand, constant self-efficacy personality traits are dynamic and changeable beliefs and may be enhanced by behavioral interventions. Successful interventions in puberty health self-efficacy are also associated with increased self-care behaviors. Therefore, self-efficacy seems to be an important and effective precondition for successful self-care behaviors in adolescents [28].. It is recommended that training sessions for adolescent girls be conducted and that interventions such as self-efficacy interventions be undertaken to promote adolescent health.

In this study, reinforces and enablers are one of the important factors for performing or not performing self-care behavior. In this study, enablers such as educational resources and training classes, appropriate sports environment for physical activity and healthy eating patterns were evaluated at a poor level. According to the findings, enablers are a predictor of behavior and has a direct and meaningful correlation with it. Hushmati and Mazloumi’s studies also showed that enablers are effective in shaping good behavior. The reinforcing factors in this study included the effects of family, friends and peers on poor health behaviors. In other studies [29, 30].

Limitations of this study are the mental state of the units under study that may influence their response. These conditions were not controlled by the researchers.

## Conclusion

The results showed that mothers’ education and other specialized educational facilities should be used such as school health care providers, education officials used the public media with cultural and educational sensitivities. And, if possible, provide counseling services for adolescents. Knowledge should not be confined to the mechanisms of puberty, menstruation, and natural behaviors, but should also help adolescents to understand more profoundly about physical changes and their relationship to sexual and health issues related to marriage due to cultural, social, and age differences. Make a huge and deep investment. Also emphasize family-based educational approaches.

## Data Availability

Authors report that the data supporting their findings can be publicly shared.

## Abbreviations

ENA: Educational needs assessment
PH: Puberty health

## References

[1] Igras SM, Macieira M, Murphy E, Lundgren R (2014). Investing in very young adolescents’ sexual and reproductive health. Glob Public Health.

[2] World Health Organization, Geneva. Health for the world’s adolescents. 2015. Available at: http://www.who.int/mediacentre/news/releases/2014/focus-adolescent-health/en/. Accessed 6 Oct 2019.

[3] Reshadat S, Zangeneh A, Saeidi S, Izadi N, Ghasemi SR, Rajabi-Gilan N (2018). A feasibility study of implementing the policies on increasing birth rate with an emphasis on socio-economic status: a case study of Kermanshah Metropolis, western Iran. Soc Indic Res.

[4] Shariaty B, Hatamy H (2013). Text Book of Public Health.

[5] Day FR, Elks CE, Murray A, Ong KK, Perry JR (2015). Puberty timing associated with diabetes, cardiovascular disease and also diverse health outcomes in men and women: the UK biobank study. Sci Reports.

[6] Zare E, Simbar M, Shahhosseini Z, Alavi MH (2017). The priorities of Iranian male adolescents health needs. Am JMens Health.

[7] Majlessi F, Rahimi A, Mahmoudi M, Hosseinzadeh P (2012). The impact of lecture and educational package methods in knowledge and attitude of teenage girls on puberty health. Hormozgan Med J.

[8] Darabi F, Yaseri M, Kaveh MH, Khalajabadi Farahani F, Majlessi F, Shojaeizadeh D (2017). The effect of a theory of planned behavior-based educational intervention on sexual and reproductive health in Iranian adolescent girls: a randomized controlled trial. J Res Health Sci.

[9] MacKenzie M (2008). Effective teaching-the design and delivery of community lectures are integral to successful adult health education programs.

[10] George N, Johnson AR, Lobo A, Pousiya S, Agrawal T (2018). Health problems and health seeking behavior among school-going adolescents in a rural area in South Karnataka. J Indian Associat ChildAdol Mental Health.

[11] Rezasefat Balesbaneh A, Mirhaghjou N, Jafsri Asl M, Kohmanaee S, Kazemnejad Leili E, Monfared A (2014). Correlation between self-care and self-efficacy in adolescents with type 1 diabetes. J Holist Nurs Mid.

[12] Mitchell C, Reid-Walsh J, Pithouse K (2004). ‘And what are you reading, miss? Oh, it is only a website’: the new media and the pedagogical possibilities of digital culture as a south African ‘Teen Guide’ to HIV/AIDS and STDs. Convergence..

[13] Kutcher S, Wei Y, McLuckie A, Bullock L (2013). Educator mental health literacy: a programme evaluation of the teacher training education on the mental health & high school curriculum guide. Advan School Mental Health Promot.

[14] Zangeneh A, Najafi F, Karimi S, Saeidi S, Izadi N (2018). Spatial-temporal cluster analysis of mortality from road traffic injuries using geographic information systems in west of Iran during 2009–2014. J foren legal Med.

[15] Cohen GL, Prinstein MJ (2006). Peer contagion of aggression and health risk behavior among adolescent males: an experimental investigation of effects on public conduct and private attitudes. Child developt.

[16] Golchin NAH, Hamzehgardeshi Z, Fakhri M, Hamzehgardeshi L (2012). The experience of puberty in Iranian adolescent girls: a qualitative content analysis. BMC Public Health.

[17] Mohammadi M, Ziapoor A, Mahboubi M, Faroukhi A, Amani N, Hydarpour F (2014). Performance evaluation of hospitals under supervision of Kermanshah medical sciences using pabonlasoty diagram of a five-year period (2008-2012). Life Sci J.

[18] Reshadat S, Zangeneh A, Saeidi S, Khademi N, Izadi N, Ghasemi SR (2016). The spatial clustering analysis of HIV and poverty through GIS in the metropolis of Kermansha, western Iran. Acta Med Mediterranea.

[19] Kheirollahi F, Rahimi Z, Arsang-Jang S, Sharifirad G, Sarraf P, Gharlipour Z (2017). Puberty health status among adolescent girls: a model-based educational program. Int J Pediatr.

[20] Brooks-Gunn J, Peterson AC. Girls at puberty: biological and psychosocial perspectives. Amazon Best Sellers Rank: Springer Science & Business Media; 2013.

[21] Afsari A, Valizadeh S, Fatahi S, Abbasnezhad M, Assdollahi M (2017). Predictors of knowledge and practice of girl students about puberty health. Intl J Pediatr.

[22] Reshadat S, Saedi S, Zangeneh A, Amooie MR, Karbasi A (2014). Equity in access to health care using geographic information system: a Kermanshah case study. J Mazandaran Univ Med Sci.

[23] Dabiri F, Abedini C, Shahi E, Kamjo A (2008). Compare the effect of education on knowledge and practice of female students Abas Bandar city in menstrual hygiene. J Hormozgan Univ Med Sci.

[24] Najafi F, Mozafari S, Mirzaee S (2012). Assessment of 3rd grade junior school girl students’ knowledge and attitude toward puberty age sanitation. J Guilan Univ Med Sci.

[25] Simbar M, Tehrani F, Hashemi Z (2005). Reproductive health knowledge, attitudes and practices of Iranian college students.

[26] De Graaf H, Vanwesenbeeck I, Meijer S, Woertman L, Meeus W (2009). Sexual trajectories during adolescence: relation to demographic characteristics and sexual risk. Arch Sex Behav.

[27] Eslamimehr F, Rakhshani F, Ramezan Khani A, Khodakarim S (2017). The examination of the effectiveness of an educational intervention based on the planned behavior theory on improving pubertal health behavior in female high school students. Int J Pediatr.

[28] Ali TS, Azam Ali P, Waheed H, Memon AA (2006). Understanding of puberty and related health problems among female adolescents in Karachi. Pakistan J Pakistan Med Associat.

[29] Mazloomymahmoodabad S, Masoudy G, Fallahzadeh H, Jalili Z (2014). Education based on precede-proceed on quality of life in elderly. Glob JHealth Sci.

[30] Valizade R, Taymoori P, Yousefi FY, Rahimi L, Ghaderi N (2016). The effect of puberty health education based on health belief model on health behaviors and preventive among teen boys in Marivan, north west of Iran. Int J Pediatr.

